# Psychopathological Symptom Load and Distinguishable Cerebral Blood Flow Velocity Patterns in Patients With Schizophrenia and Healthy Controls: A Functional Transcranial Doppler Study

**DOI:** 10.3389/fpsyt.2021.679021

**Published:** 2021-06-25

**Authors:** Stephan T. Egger, Julio Bobes, Katrin Rauen, Erich Seifritz, Stefan Vetter, Daniel Schuepbach

**Affiliations:** ^1^Department of Psychiatry, Psychotherapy and Psychosomatics, Faculty of Medicine, University Hospital of Zurich, University of Zürich, Zurich, Switzerland; ^2^Department of Psychiatry, Faculty of Medicine, University of Oviedo, Oviedo, Spain; ^3^Department of Geriatric Psychiatry, Faculty of Medicine, Psychiatric University Hospital of Zurich, University of Zürich, Zurich, Switzerland; ^4^Institute for Stroke and Dementia Research, Ludwig Maximilian University Munich, University Hospital, Munich, Germany; ^5^Department of General Psychiatry, Center of Psychosocial Medicine, University of Heidelberg, Heidelberg, Germany; ^6^Klinikum am Weissenhof, Weinsberg, Germany

**Keywords:** transcranial doppler, schizophrenia, symptomalogy, trail making test, cognition, hemodynamics

## Abstract

**Introduction:** Schizophrenia is a severe psychiatric disorder, with executive dysfunction and impaired processing speed playing a pivotal role in the course of the disease. In patients with schizophrenia, neurocognitive deficits appear to be related to alterations in cerebral hemodynamics. It is not fully understood if psychopathological symptom load (i.e., presence and severity of symptoms) is also related to alterations in cerebral hemodynamics. We aim to study the relationship between psychopathological symptom load and cerebral hemodynamics in the Middle Cerebral Artery (MCA) during a cognitive task in patients with schizophrenia and healthy controls.

**Methodology:** Cerebral hemodynamics in the MCA were examined in 30 patients with schizophrenia and 15 healthy controls using functional Transcranial Doppler (fTCD) during the Trail Making Test (TMT). Psychopathological symptoms were measured using the Brief Psychiatric Rating Scale (BPRS). Patients were dichotomized according to BPRS scores: mild-moderate (BPRS < 41, *n* = 15) or marked-severe (BPRS ≧ 41, *n* = 15). Mean blood flow velocity (MFV) in the MCA and processing speed of the TMT were analyzed. Cerebral hemodynamics were analyzed using the general additional model (GAM) with a covariate analysis of variance (ANCOVA) for group comparisons.

**Results:** Patients and healthy controls were comparable regarding demographics. Patients had a slower processing speed for the TMT-A (patients-severe: 52s, patients-moderate: 40s, healthy-controls: 32s, *p* = 0.019) and TMT-B [patients-severe: 111s, patients-moderate: 76s, healthy-controls: 66s, *p* < 0.001)]. Patients demonstrated differing hemodynamic profiles in both TMTs: TMT- A [*F*_(6, 1,792)_ = 17, *p* < 0.000); TMT-B [*F*_(6, 2,692)_ = 61.93, *p* < 0.000], with a delay in increase in MFV and a failure to return to baseline values.

**Conclusions:** Patients with schizophrenia demonstrated slower speeds of processing during both the TMT-A and TMT-B. The speed of processing deteriorated with increasing psychopathological symptom load, additionally a distinct cerebral hemodynamic pattern in the MCA was observed. Our results further support the view that severity of schizophrenia, particularly psychopathological symptom load, influences performance in neurocognitive tasks and is related to distinct patterns of brain hemodynamics.

## Introduction

Schizophrenia is a severe psychiatric disorder which is characterized by hallucinations, delusions and blunted affect (1). Although not part of the diagnostic criteria, cognitive impairment is also a common feature of schizophrenia, often occurring before the onset of the first psychotic episode and continuing throughout the course of the disease (2, 3), with cognitive impairment and executive functions playing a pivotal role for outcome and prognosis, as well as being major determinants of quality of life and well-being (4, 5).

Cognitive impairment and executive function are measured by numerous neuropsychological assessment tools, including the Trail Making Test (TMT)-A and TMT-B. In contrast to other neuropsychological instruments, the TMTs are easy to use; their interpretation is straightforward. Consequently, they are widely used in both research and clinical practice (6, 7). The TMTs are considered to be sensitive to cognitive dysfunction and frontal lobe integrity, assessing graphomotor activity, visual scanning, selective attention, mental flexibility and executive functioning (6, 7). Patients with schizophrenia show impaired performance in the TMT, with deficits in processing speed and inefficient simultaneous processing strategies (6, 8). There is evidence that in patients with schizophrenia, the frontal lobes, particularly the dorsolateral prefrontal cortex (DLPFC), play a pivotal role in executing the TMTs (9, 10).

Cognitive performance is partly determined by the brain's ability to increase blood supply to the areas activated during a cognitive task. Due to the skull's anatomic conditions, the increase in diameter of the cerebral arteries is limited; an increase in cerebral blood supply is achieved by increasing blood flow velocity in the cerebral arteries. Therefore, we consider Mean Flow Velocity (MFV) a valid indicator for brain activity (11, 12). Transcranial Doppler (TCD) is a versatile, non-invasive method for assessing the cerebral arteries' functioning and hemodynamic characteristics (11, 13). TCD provides a continuous measurement of blood flow velocity with high temporal resolution. However, TCD has low anatomical resolution and is not able to deliver a direct brain image. Despite this limitation, TCD has been used to study physiological and hemodynamic conditions in several neurological diseases and psychiatric disorders (11, 13, 14). The neurocognitive impairment in schizophrenia appears to be related to alterations in blood flow in several brain areas, including the DLPFC (15–18).

The middle cerebral arteries (MCA) irrigate the brain's lateral hemispheres, including the DLPFC, subcortical structures, basal ganglia and the striatum (19). A number of these structures, principally the DLPFC, striatum and thalamus, are activated during the TMT (17), making fTCD a suitable method for the physiological and hemodynamic assessment of brain activity in these areas during a neurocognitive task (20). Previous studies using functional TCD (fTCD) in patients with schizophrenia and healthy controls showed clear differences in the MCA hemodynamic pattern during the TMT (14); to what extent there are also hemodynamic differences between those affected with schizophrenia, in relation to the number and severity of symptoms (i.e., symptom load) is thus far unexplored.

Our study aims to determine the relationship between psychopathological symptom load (in healthy controls and patients with schizophrenia) and cerebral hemodynamics in the MCA during a neurocognitive task. New results may contribute to increased use of fTCD as an assessment tool in neuropsychiatric disorders, particularly schizophrenia. We examined cerebral blood flow velocity during the TMT in patients with schizophrenia and healthy controls, using a visuomotor control task to compensate for hemodynamic changes resulting solely from the motor and visual activities during the TMT.

## Materials and Methods

### Subjects

Thirty patients fulfilling the WHO-ICD 10 (21) criteria for schizophrenia participated in this study; they were age and sex-matched with 15 healthy controls. The healthy controls had no medical, neurological or psychiatric condition at the time of examination; they were recruited for a previous study conducted by our research group, using the same examination protocol and equipment (14). All participants were right-handed. All patients with schizophrenia were taking antipsychotic medication. The following exclusion criteria applied to patients: 1. affective disorder (according to ICD-10: F3); 2. organic brain disorder (according to ICD-10: F0); 3. active substance abuse disorder (according to ICD-10: F1) in the 3 months before inclusion; 4. unstable neurological; or 5. medical condition. Basic demographic characteristics of the participants were collected. Besides the participants' education, we included the mean education of their parents to disentangle poor educational performance attributable to early onset of the disorder or familial accumulation. The competent ethics committee approved the study, all participants provided written informed consent.

### Clinical Assessment and Psychometric Measurements

Within 24 h of the fTCD measurement, psychopathological symptoms were assessed using the Brief Psychiatric Rating Scale (BPRS) (22), overall clinical severity of symptoms was assessed using the Clinical Global Impression Scale (CGI) (23). For this study, the daily antipsychotic dose was converted to chlorpromazine equivalents according to current guidelines (24, 25). Effects of antipsychotics on the extrapyramidal system were examined using the Simpson- Angus Scale (SAS) (26) and the Barnes Akathisia Scale (BAS) (27, 28).

The BPRS is one of the most frequently used scales to measure psychopathology in patients with schizophrenia, systematically assessing the presence and severity of symptoms. It consists of 18 single items assessing different symptoms. Each item is evaluated according to a seven-item Likert scale, ranging from “1” (not present) to “7” (extremely severe). Thus, the sum score ranges from 18 to 126. We used the BPRS sum score as a measure of the psychopathological symptom load. Participants were classified according to BPRS sum scores, as “non-affected” or healthy controls; those with a diagnosis of schizophrenia and a BPRS score below 41 points were classified as “mild-moderate,” those with a BPRS score of 41 points or more were classified as “marked- severe”(29).

### Equipment and Cerebral Blood Flow Measurements

Doppler measurements were performed using a Multi-Dop X instrument (DWL Elektronische Systeme GmbH, Sipplingen- Germany). Two dual 2 MHz transducers were attached and fixed with a headband. Both MCAs were insonated at depths of 48–55 mm through the temporal bone window. The 2 MHz transducers were fixed with a headband, so motion artifacts of the head did not alter the position of the transducers. This approach is supported by published evidence demonstrating that functional transcranial Doppler is fairly robust to movement artifacts (30). As indicated by measurement artifact data, we screened for MFV values outside the 60–150% range of the mean MFV recording of a subject before, after and during the cognitive task.

### Cerebral Hemodynamics and Cognitive Task

Subjects were asked to abstain from caffeine and nicotine consumption 2 h prior to the examination (31). MFV data were continuously recorded during the psychological paradigm, integrating MFV data for each cardiac cycle. Participants underwent a standardized briefing. They were instructed about the nature of the study and the psychological paradigm. To reduce learning effects, the cognitive task was presented only once. We administered the TMTs as a paper and pencil test. In the TMT-A, subjects had to connect 25 numbers in ascending order (i.e., 1, 2, 3,..., 25). In the TMT-B, participants had to connect numbers (1–13) and letters (A–L) alternately in ascending order (i.e., 1, A, 2, B, 3, C,..., 13, L). Subjects had to solve the TMTs as quickly and accurately as possible. In the control task participants were asked to randomly connect circles placed in a 10 by 10 cm square. Lines had to be drawn at a pace of 1.0 or 0.5 Hz to simulate the pace of the TMT-A (0.89+−0.21 Hz) and the TMT-B (0.46+−0.15Hz). The control task simulates visuomotor scanning during the TMTs. The control task was placed randomly before or after each TMT, with a break of 60 s between each task.

### Statistical Analysis

Data are presented in tables using simple descriptive statistics (mean, standard deviation, percentages). For the analysis of group differences, specific statistical tests were performed. Continuous data were analyzed using a univariate analysis of variance (ANOVA), with a secondary *t*-test to evaluate model differences. The chi-square test was applied to categorical data. A *post hoc* power analysis was conducted, using the effect sizes for differences in completion time between the TMT-A and TMT-B.

For the purposes of analysis, the MFV consisted of the following elements, following procedures used in a previous study (20): (a). integration of MFV from 100 Hz sampling to 1 Hz; (b). normalization of digitized data with reference to pre-and post-task rest phases (60s intervals of rest with 30s between the first and last 15s); and (c) relative MFV (relative to resting state) values, averaged and converted to percentage values. All MFV values in this paper are relative MFV, i.e., cerebral blood flow velocity change compared with resting phase values. For analysis of the TMT-A and B, the time to be analyzed was dictated by the time required by the fastest participant to complete the task.

The general additional model (GAM); was used for graphical representation, as well as to statistically evaluate the change in mean flow velocity over time (in seconds), controlled for side and sex. The advantage of non-parametric tests, such as the general additional model, lies in their greater flexibility regarding assumptions about data (32–34). The GAM allows for regression and weight analysis at both fixed and random variable level (or for discrete and continuous variables) (33). Using a non-parametric test allows for a realistic visual comparison of flow velocity, facilitating the inference of its clinical relevance (35, 36). Accordingly, a better representation of dynamic and inter-dependent results such as blood flow is provided. However, the mathematical and statistical analysis and consequently, comparison of the GAMs outcomes is more complex (34, 37). Therefore, a covariate analysis of variance (ANCOVA) was used to evaluate differences in the GAM of blood flow velocity obtained for each group and side. Thus, allowing us to determine whether a statistical difference between the hemodynamic curves was demonstrated., A pairwise-comparison was conducted to determine the time frames in which the curves differed from one another.

## Results

### Demographics and Clinical Characteristics

Patients and control subjects were comparable regarding age, sex and years of parent's education. Years of own education for those with schizophrenia was significantly shorter than healthy controls, with no difference between severity groups. Patients with a marked-severe psychopathological symptom load obtained significantly higher CGI-S scores than those with moderate symptomatology [3.60 ± 1.06 vs. 5.20 ± 0.68, *F*_(1, 28)_ = 24.44, *p* < 0.001]. The duration of illness and hospitalization rates did not differ significantly between patient groups. Each participant with a diagnosis of schizophrenia had an antipsychotic prescribed, some two. Most antipsychotics prescribed were second-generation antipsychotics. There were no significant differences regarding the antipsychotics prescribed (data not shown) and dose (as chlorpromazine equivalents). Furthermore, the rate of extrapyramidal motor symptoms and akathisia was also similar (see Table 1). The *post hoc* power analysis reached a power of 1-ß of 0.99.

**Table 1:** Demographic and clinical characteristics of the sample.

	**Non- affected**	**Mild-moderate**	**Marked-severe**		
	***N* = 15**	***N* = 15**	***N* = 15**		
**Demographic Variables**				Statistics	*p*
Age (in years)	33.87 (7.68)	34.05 (7.73)	32.40 (5.38)	*F*_(2, 42)_ = 0.222	0.80
Sex (male/female)	10/5	10/5	12/3	${\text{X}}_{\left(2,\;45\right)}^{2}$ = 0.865	0.65
Education (in years)	19.17 (4.17)^a^	13.07 (2.60)^a^	13.10 (2.48)^a^	*F*_(2, 42)_ = 18.313	<0.001
Parents‘ Education (in years)	15.47 (3.83)	14.70 (3.00)	13.97 (3.03)	*F*_(2, 42)_ = 0.769	0.47
**Clinical variables**					
BPRS	–	30.27 (4.91)^b^	43.73 (3.67)^b^	*F*_(1, 28)_ = 72.421	<0.001
CGI-S	–	3.60 (1.06)^b^	5.20 (0.68)^b^	*F*_(1, 28)_ = 24.436	<0.001
Chlorpromazine equivalent dosage (mg/d)		531.67 (165.69)	543.33 (176.14)	*F*_(1, 28)_ = 0.035	0.85
EPS	–	3.47 (2.10)	4.93(7.91)	*F*_(1, 28)_ = 0.481	0.49
BAS	–	0.53 (0.74)	1.07 (0.96)	*F*_(1, 28)_ = 2.89	0.10
Duration of illness (in years)	–	8.67 (6.91)	12.20 (4.04)	*F*_(1, 28)_ = 2.922	0.09
Number of hospitalizations	–	3.47 (2.03)	5.13 (4.70)	*F*_(1, 28)_ = 1.587	0.22
**Cognitive performance**					
TMT-A (duration in seconds)	31.00 (7.43)^c^	40.20 (12.66)^c^	52.27 (30.84)^c^	*F*_(2, 42)_ = 4.388	0.019
TMT-B (duration in seconds)	66.20 (18.89)^c^	75.53 (22.98)^c^	111.13 (20.99)^c^	*F*_(2, 42)_ = 19.085	<0.001

*Pairwise comparison:*

a *non-affected > mild-moderate and marked-severe;*

b *marked-severe > mild-moderate:*

c *marked-severe < mild-moderate < non-affected*.

### TMT Performance

In comparison to healthy controls, patients with schizophrenia required significantly more time to complete both TMTs. Furthermore, more severely ill patients took significantly longer to complete the test than those classified as mild-moderately ill; TMT-A [patients-severe: 52.3 ± 30.8; patients-moderate: 40.2 ± 12.7; healthy-controls: 31.0 ± 7.4, *F*_(2, 42)_ = 4.38, *p* = 0.019] and TMT-B [patients-severe: 66.2 ± 18.9; patients-moderate: 75.5 ± 22.9; healthy-controls: 111.1 ± 20.9, *F*_(2, 42)_ = 19.08, *p* < 0.001]. Since the fastest performance on the TMT-A was 20s, and for the TMT-B 30s, these are the time periods considered for statistical analysis. There was no statistically significant difference between groups regarding the rate of errors on the TMTs (see Table 1).

### Mean Cerebral Blood Flow Velocity During the TMT-A

For healthy-controls, there was a significant change in MFV over time [*F*(s)_(3.991, 4.912)_ = 15.43, *p* < 0.001], with a hemispheric difference in MFV [*F*_(1, 266)_ = 10.55, *p* = 001]; in the *post hoc* pairwise analysis we identified that the hemispheric (right > left) difference was only significant for the first 10 s of the measurement period. For those mildly-moderately affected, there was also a change of MFV over time [*F*(s)_(7.403, 8.356)_ = 6.707, *p* < 0.001], we did not find a hemispheric difference in the MFV [*F*_(1, 266)_ = 0.979, *p* = 0.323]; with the *post hoc* pairwise analysis also demonstrating no hemispheric differences at any time point. Finally, in those with higher psychopathological symptom load, we found a change in MFV over time [*F*(s)_(8.087, 8.767)_ = 9.746, *P* < 0.001], with a hemispheric difference (left > right) in the MFV [*F*_(1, 266)_ = 7.71, *p* < 0.001]; the *post hoc* pairwise comparison indicating that this difference was only significant during the middle phase of the measurement (s 9 to 14). There is a statistically significant difference between the curves of the three groups under comparison [*F*_(6, 1,792)_ = 17, *p* < 0.000].

**Figure 1:**
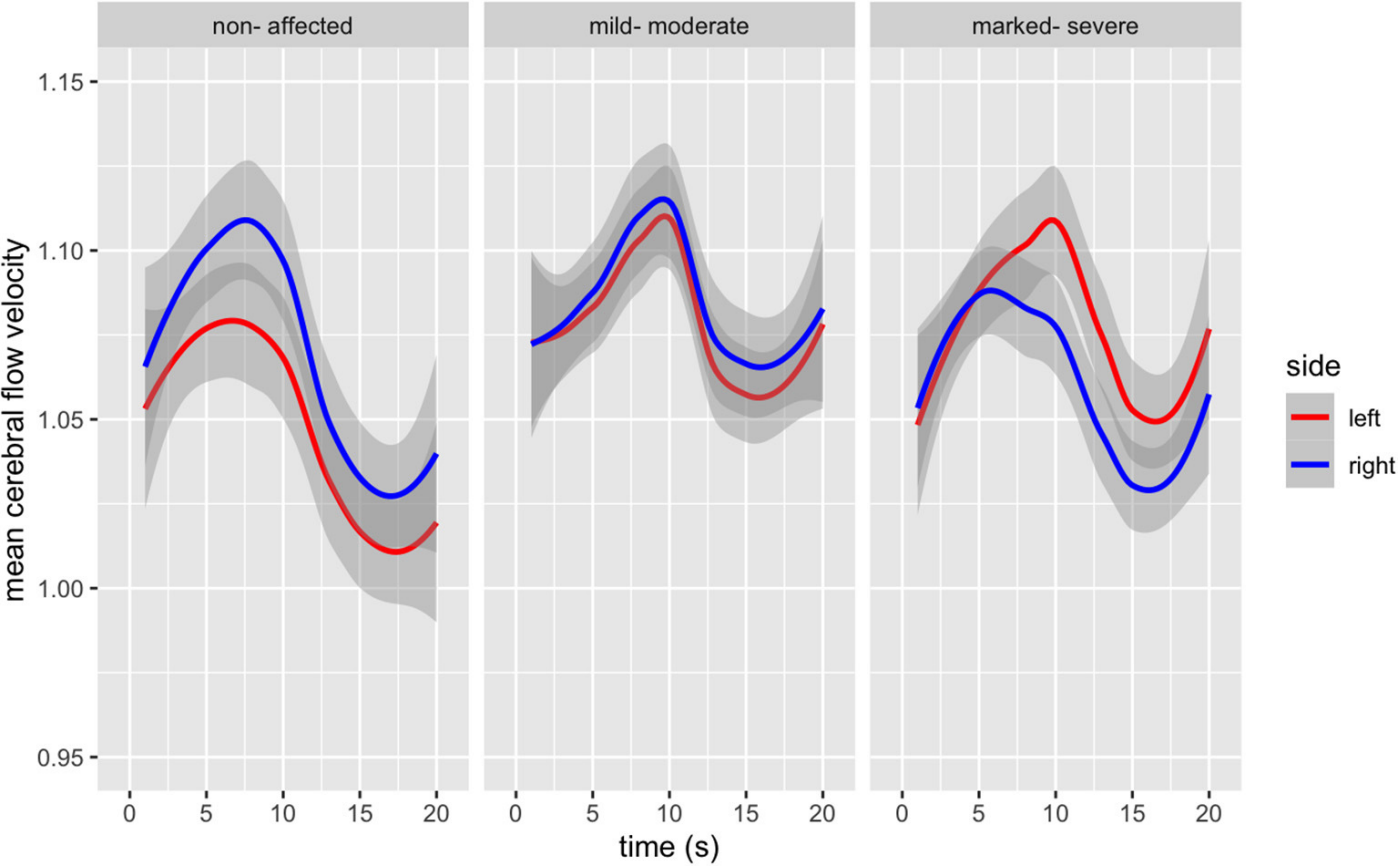
Bilateral Mean Flow Velocity changes from baseline in the Middle Cerebral Artery during the duration of the TMT-A (in seconds), according to psychopathological symptom load [*F*_(6, 1,792)_ = 17, *p* < 0.000].

Group differences and hemispheric differences in MFV during the TMT-A are graphically represented (Figure 1). The mean flow velocity in both middle cerebral arteries shows a similar pattern for all three groups during the TMT-A; in the first 5–10 s, there is an increase in blood flow followed by a steady decrease. Healthy controls reached the peak of blood flow 2 s faster than those with schizophrenia. Furthermore, those with marked-severe psychopathological symptom load show a delayed and higher increase and a slighter decrease in the blood blow velocity in the left MCA.

### Mean Cerebral Blood Flow Velocity During the TMT-B

Healthy controls demonstrate a change of MFV over time [*F*(s)_(4.890, 5.952)_ = 23.04, *p* < 0.001], without a hemispheric difference in MFV [*F*_(1, 406)_ = 0.693, *p* = 0.405] during the TMT-B. Those mild-moderately affected also showed a change in MFV over time [*F*(s)_(8.106, 8.779)_ = 18.62, *p* < 0.001], without a hemispheric difference in MFV [*F*_(1, 406)_ = 1.117, *p* = 0.291], the *post hoc* pairwise comparison, however, demonstrated a difference for the first eight s of the measurement period. Those more severely affected also demonstrated a change in MFV over time [*F*(s)_(5.750, 6.882)_ = 3.888, *p* < 0.001], and a hemispheric difference in MFV [*F*_(1, 406)_ = 28.42, *p* < 0.001]; with the pairwise *post hoc* comparison revealing a significant difference for the first 5 s and in the middle phase of the measurement period (s 18 to 21). There is a statistically significant difference between the curves of the three comparison groups [*F*_(6, 2,692)_ = 61.93, *p* < 0.000).

**Figure 2:**
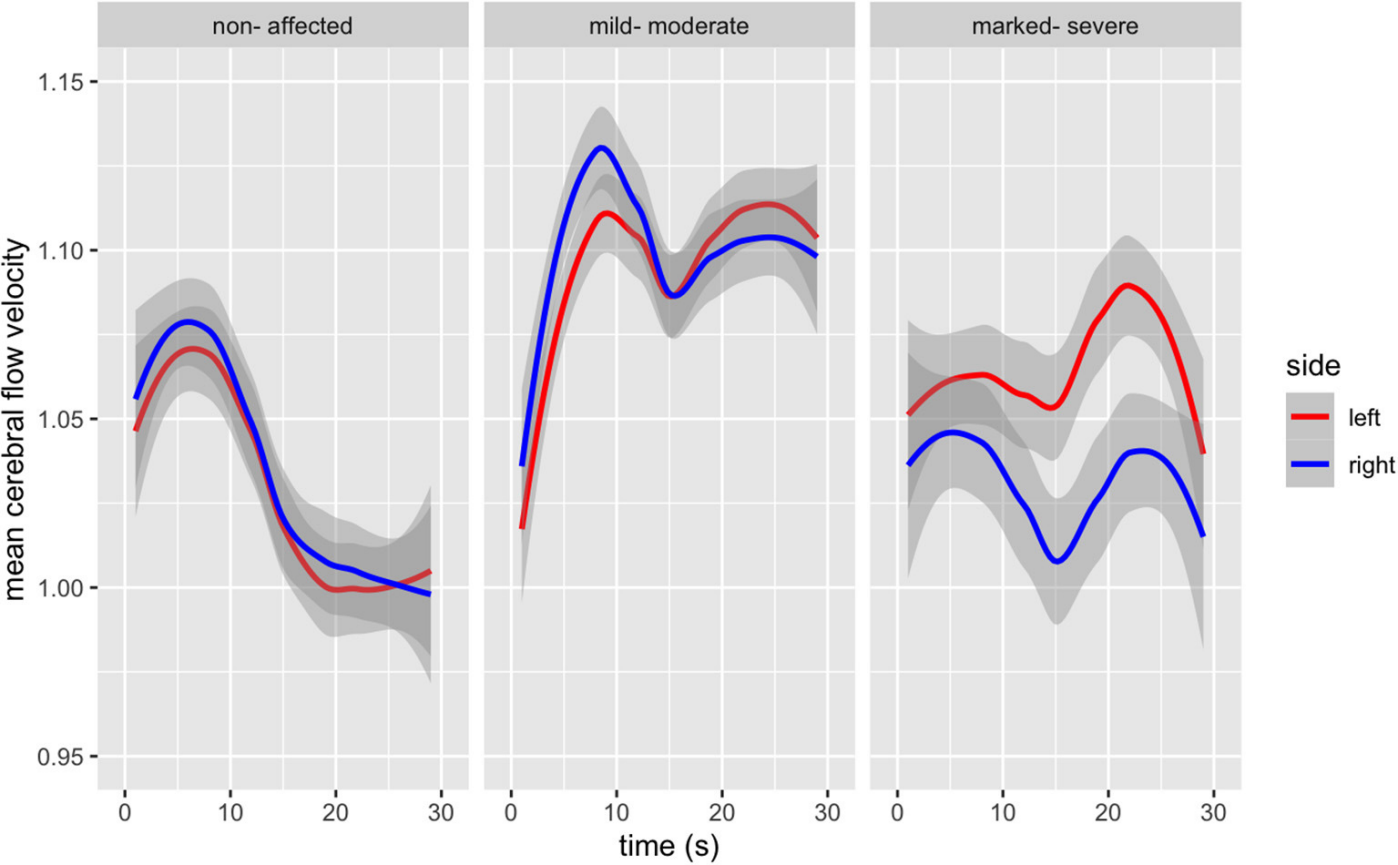
Bilateral Mean Flow Velocity changes from baseline in the Middle Cerebral Artery during the duration of the TMT-B (in seconds), according to psychopathological symptom load [*F*_(6, 2,692)_ = 61.93, *p* < 0.000].

Group differences and hemispheric differences in MFV during the TMT-B are graphically represented (Figure 2). Healthy controls reach a peak in blood flow after 5 s, with a continuous decrease. Those with mild-moderate psychopathological symptom load also reach the first peak after 7 s, followed by a slight decrease and a second lower peak. Finally, those with more severe schizophrenia show a discrepancy between both MCAs. Both MCAs form two peaks, the first just a few seconds after beginning the task, the second after 20 s. The right MCA shows a lower increased MFV, demonstrating a lower initial peak than the left MCA 20 s after beginning the task.

## Discussion

Using an age and gender-balanced sample population, including patients with schizophrenia and healthy controls, we examined the mean blood flow velocity during the TMT-A and TMT-B in the middle cerebral artery using functional transcranial doppler. In our study, participants with a higher psychopathological symptom load (represented by higher BPRS scores) demonstrated slower processing speed (with similar accuracy) during the TMT-A and TMT-B, compared with participants with fewer symptoms and healthy controls. The hemodynamic pattern also demonstrated a clearly distinguishable profile between patients with schizophrenia and healthy controls. The differences were more marked in those with higher symptom severity and when the complexity of the cognitive task increased (i.e., TMT-B over TMT-A). In summary, our results demonstrate a relationship between psychopathological symptom load, cognitive demand, decreased processing speed and distinct hemodynamic patterns in the MCA during the TMT-A and TMT-B.

Healthy controls in our study demonstrate in both the TMT-A and TMT-B, an initial increase in cerebral blood flow, which returns smoothly to baseline values, thus reproducing previous findings in healthy subjects (38). Patients with schizophrenia fail to reproduce this pattern. Firstly, the increase in MFV is delayed; secondly, the MFV fails to return to baseline values. The differences in the hemodynamic pattern for patients with schizophrenia are accentuated, as psychopathological symptom load and cognitive demand increases (i.e., TMT-B over TMT-A). Processing speed may mediate the demonstrated differences in hemodynamic pattern according to symptom severity and cognitive demand for the TMT-A and TMT-B. This lends further support to the notion that neuronal activity and cerebral blood flow are closely coupled; with MFV resulting from the activation of (or correspondingly the failure to deactivate) cortical areas in the MCA's irrigation territory (9, 12, 19, 39, 40). Patients with schizophrenia demonstrate increased brain activity (as indicated by a higher MFV) for lower performance (i.e., slower processing speed) compared to healthy controls (16).

Neuroimaging studies demonstrate different brain activation patterns for the TMT-A and TMT-B. In the case of the TMT-A, areas involved in graphomotor speed, visual scanning, and selective attention are activated, whereas for the TMT-B, activated areas relate to mental flexibility and executive functioning (8). Previous findings also report a positive correlation between the TMT-B and hemodynamic activity in the MCA; this may be due to activation of the DLPFC, temporal cortex, basal ganglia and the thalamus during the TMT-B (17, 18). Several of these neuroanatomical areas are also involved in psychopathology and the neurocognitive anomalies related to schizophrenia (14, 41). Studies (including ours) using fTCD demonstrate a correlation between performance and hemodynamic patterns (14–16). This lends support to the view that factors inherent to schizophrenia as well as to other conditions characterized by executive dysfunction, such as dysfunctional neuronal integrity, accelerated white matter aging, hypoperfusion and increased vascular resistance (42–45) may play a role. Since our sample was matched for age and gender, we cannot make any inferences regarding the effects of aging on our results. In the absence of a direct anatomical image, taking into account that the irrigation territory of the MCA is extensive, our findings in this respect are not conclusive.

Our study has some other limitations which must be taken into account in order to better understand and interpret our findings. The lack of further neurocognitive assessments limits our results to the cognitive abilities measured by the TMTs. Taking into account that patients and healthy controls showed similar error rates (i.e., both were accurate), the main difference between the subsamples is processing speed. Medication, particularly antipsychotics, can impair cognitive performance, whether directly or through side effects (46–50), Furthermore, they can also influence hemodynamics (47). In our design, we did not directly control for this potentially confounding factor. Nonetheless, we did not find a difference regarding the dose and side effects of antipsychotics among patients with schizophrenia. Those with a higher psychopathological symptom load had a longer course of the disease with higher rates of hospitalization, which may be related to more severely impaired cognitive performance (51, 52). The influence of systemic circulation on cerebral blood flow, especially heart rate and arterial blood pressure, is controlled for using a random motor activity (53, 54). The control task aims to compensate for subtle alterations in circulation and other confounding factors through relative values compared to resting phase values before and after the paradigm (53). Furthermore, all participants were medically and neurologically stable, with no circulatory anomalies at the time of the study. Other potential confounders, such as anxiety, hypo- or hyperventilation (55), were not observed during the measurement procedure.

In summary, patients with schizophrenia performed less satisfactorily on both TMTs. Performance deteriorated with increasing symptom load, parallel with a distinct cerebral blood flow pattern in the MCA. Our results further support the view that schizophrenia, particularly symptom load and thus severity, influences performance in neurocognitive tasks, whilst being related to distinct brain hemodynamic patterns. Furthermore, these results support the use of fTCD as a brain imaging technique capable of studying brain hemodynamics during neurocognitive tasks.

## Data Availability Statement

The raw data supporting the conclusions of this article will be made available by the authors, without undue reservation.

## Ethics Statement

The studies involving human participants were reviewed and approved by Kantonale Ethikkommission of Zurich. The patients/participants provided their written informed consent to participate in this study.

## Author Contributions

SE and DS conceived the presented idea, carried out the experiment, and analyzed the results and prepared the manuscript. JB and ES aided in interpreting the results and supervised the manuscript. KR and SV contributed to the interpretation of the results. All authors contributed to the article and approved the submitted version.

## Conflict of Interest

The authors declare that the research was conducted in the absence of any commercial or financial relationships that could be construed as a potential conflict of interest.
